# Morphological Characteristics of Idiopathic Inflammatory Myopathies in Juvenile Patients

**DOI:** 10.3390/cells11010109

**Published:** 2021-12-30

**Authors:** Anne Schänzer, Leonie Rager, Iris Dahlhaus, Carsten Dittmayer, Corinna Preusse, Adela Della Marina, Hans-Hilmar Goebel, Andreas Hahn, Werner Stenzel

**Affiliations:** 1 Institute of Neuropathology, Justus Liebig University, 35392 Giessen, Germany; leonie.j.rager@med.uni-giessen.de; 2 Institute of Medical Informatics, Charité—Universitätsmedizin Berlin, Corporate Member of Freie Universität Berlin und Humboldt-Universität zu Berlin, 10117 Berlin, Germany; iris.dahlhaus@charite.de; 3 Department of Neuropathology, Charité—Universitätsmedizin Berlin, Corporate Member of Freie Universität Berlin and Humboldt-Universität zu Berlin, 10117 Berlin, Germany; carsten.dittmayer@charite.de (C.D.); corinna.preusse@charite.de (C.P.); hans-hilmar.goebel@charite.de (H.-H.G.); werner.stenzel@charite.de (W.S.); 4 Department of Neurology with Institute of Translational Neurology, University Hospital Münster, 45147 Münster, Germany; 5 Department of Pediatric Neurology, Centre for Neuromuscular Disorders, Centre for Translational Neuro-und Behavioral Sciences, University Duisburg-Essen, 45122 Essen, Germany; adela.dellamarina@uk-essen.de; 6 Department of Child Neurology, Justus Liebig University, 35392 Giessen, Germany; andreas.hahn@paediat.med.uni-giessen.de

**Keywords:** myositis, dermatomyositis, muscle pathology, overlap myositis, juvenile, anti-synthetase syndrome

## Abstract

**Background:** In juvenile idiopathic inflammatory myopathies (IIMs), morphological characteristic features of distinct subgroups are not well defined. New treatment strategies require a precise diagnosis of the subgroups in IIM, and, therefore, knowledge about the pathomorphology of juvenile IIMs is warranted. **Methods:** Muscle biopsies from 15 patients (median age 8 (range 3–17) years, 73% female) with IIM and seven controls were analyzed by standard methods, immunohistochemistry, and transmission electron microscopy (TEM). Detailed clinical and laboratory data were accessed retrospectively. **Results:** Proximal muscle weakness and skin symptoms were the main clinical symptoms. Dermatomyositis (DM) was diagnosed in 9/15, antisynthetase syndrome (ASyS) in 4/15, and overlap myositis (OM) in 2/15. Analysis of skeletal muscle tissues showed inflammatory cells and diffuse upregulation of MHC class I in all subtypes. Morphological key findings were COX-deficient fibers as a striking pathology in DM and perimysial alkaline phosphatase positivity in anti-Jo-1-ASyS. Vascular staining of the type 1 IFN-surrogate marker, MxA, correlated with endothelial tubuloreticular inclusions in both groups. None of these specific morphological findings were present in anti-PL7-ASyS or OM patients. **Conclusions:** Morphological characteristics discriminate IIM subtypes in juvenile patients, emphasizing differences in aetiopathogenesis and supporting the notion of individual and targeted therapeutic strategies.

## 1. Introduction

Idiopathic inflammatory myopathy (IIM) is the most common form of myopathy in adult patients. Based on new insights in pathogenesis, it has become clear that individual immunomodulatory therapy strategies may be helpful for patients who do not respond sufficiently to current standard therapy [1,2]. Classification of IIM has been based on clinical, serological, and morphological features that lead to further definition of distinct subgroups, such as dermatomyositis (DM), antisynthetase syndrome (ASyS), immune-mediated necrotizing myopathy (IMNM), and sporadic inclusion body myositis (sIBM) [3,4,5,6]. Additionally, myositis can be associated with various rheumatologic diseases and is then named overlap myositis (OM) [7,8]. The currently used classification of myositis, based on pathophysiological and immunological insights, suggests that subtype-specific therapy may be the best treatment option [9].

Juvenile idiopathic inflammatory myopathy (jIIM) has many features in common with adult IIM. Both age groups harbor distinct clinical phenotypes, autoantibody findings, and associated outcomes [10,11,12,13]. However, a stringent classification of jIIM, including clinical, serological, and morphological features, similar to the one in adult patients, does not exist. This results in uncertainty when assigning children with myositis to different subgroups, and makes interpretation of study results and assessment of outcomes, including therapy failure, difficult. Myositis specific antibodies (MSAs) are implemented to diagnose juvenile IIM, allowing the delineation of distinct subtypes, such as DM, ASyS, and necrotizing myopathy (IMNM). Dependent on the respective studies, up to 50–90% of the patients show that MSAs with the most frequent antibodies are anti-MDA5, anti-NXP2, and anti-TIF-1γ [11,13,14,15,16].

Myositis, in conjunction with skin lesions, is also seen in other rheumatologic disorders, such as systemic lupus erythematosus (SLE), connective tissue diseases, and some monogenic autoinflammatory diseases, further impeding the diagnosis and treatment of jIIM [14,17,18,19,20]. Moreover, in jIIM patients with negative autoantibodies, finding a precise diagnosis can be problematic (although it is currently not known whether autoantibodies have the same relevance in children as in adults).

Juvenile DM is the most common form of jIIM affecting 2–4/100,000 children per year [11,21]. JDM can start as early as two years, and girls are more often affected than boys [10,22]. Clinical key features are skeletal muscle weakness with signs of inflammation on MRI and muscle biopsy, skin lesions, and systemic organ involvement. Phenotype and prognosis are highly variable [23]. Lipodystrophy and calcinosis uncommon in adult DM are frequently reported in jDM [24]. MSA can be detected in approximately 70% of patients. NXP-2 and TIF1γ antibodies are most frequent, followed by MDA5 and Mi-2 [25,26,27] antibodies. With the introduction of corticosteroids, mortality has dropped to 2–3% [13]. However, steroid resistance in some patients results in poor prognosis and warrants new therapeutic strategies [28]. IFN-related endothelial injury and Janus kinase (JAK) inhibition has emerged as a novel therapeutic strategy in difficult-to-treat cases [1,29,30].

Clinical characteristics, MSA profiles, and muscle pathology are well described in adult DM, but can be different in jDM (for review see Tanboon et al., 2020 [6]) [3,31,32]. These differences are not fully explained and may reflect different modifying factors in jDM and aDM [22]. Muscle biopsies show the morphological hallmarks of perifascicular pathology and inflammatory cells with a high variability of pathology in both adult and juvenile DM [25,26,33]. However, distinct morphological features in MSA-defined DM subgroups and association with clinical phenotypes are mainly described in aDM [11,26].

Autoantibodies against the cytoplasmatic aminoacyl-tRNA synthetase (ARS) are associated with ASyS [34,35,36]. The frequency of ARS antibodies and, hence, the anti-synthetase syndrome are much lower in children (1–3%) compared to adults (30–40%) [13,37]. Clinical manifestation is similar in all age groups often associated with interstitial pneumonia [38]. The morphological hallmark of ASyS is a necrotizing perifascicular myositis with strong staining of the fragmented perimysium in alkaline phosphatase preparations in Jo1-associated ASyS [39,40]. Additionally, myonuclear actin filament inclusions are a unique morphological feature in adult ASyS [41].

Finally, IMNM is associated with severe muscle weakness, high creatine kinase levels, no or little skin involvement, and scattered necrotic and regenerating fibers at muscle biopsy [42,43]. IMNM is paradigmatically associated with anti-HMCGR or anti-SRP autoantibodies. IMNM is rare in children accounting for 1–4% of all jIIM cases [13,14,44,45].

In this single tertiary center study, we performed a detailed semi-quantitative analysis of muscle biopsies from fifteen children diagnosed with jIIM and seven non-diseased controls based on enzymatic stains, immunohistochemistry, and electron microscopy, and compared the morphological findings with clinical and laboratory data. The aim of the study was to identify morphological key findings which discriminate the different subtypes of juvenile IIM.

## 2. Materials and Methods

### 2.1. Subjects and Samples

Fifteen juvenile patients with the clinical diagnosis of myositis were included in the study. Patients were recruited from the department of pediatric neurology, Giessen, between 2002 and 2018. An open muscle biopsy was performed on all patients. Clinical and laboratory data were retrospectively analyzed in the database. Controls were obtained from seven age-matched children, who received a muscle biopsy because of suspected neuromuscular disorder or metabolic disease confirmed by biopsy and further clinical follow-up. All control muscle biopsies did not show any overt pathological changes, including thorough studies of immune phenomena. The median age at biopsy was 12 (9–17) years, 33% female.

### 2.2. Muscle Pathology Analysis

#### 2.2.1. Histochemical, Enzymatic and Immunohistochemical Microscopy

The unfixed muscle tissue was snap-frozen, and 6-µm cryosections were processed for histochemical and enzymatic staining, according to standard procedures including hämalum eosin (H&E) combined cytochrome-c-oxidase (COX)–succinate dehydrogenase (SDH), Gömöri trichrome, and alkaline phosphatase (ALP) [46]. Immunohistochemical stains were performed on cryosections using a Bench Mark XT automatic staining platform (Ventana, Heidelberg, Germany) with the following primary antibodies: mouse monoclonal anti-LC3 (0231-100/LC3-5F10, nanoTools, 1:100, Teningen, Germany); mouse monoclonal anti-p62 (610832, BD Biosciences, 1:500, Franklin Lakes, NJ, USA); mouse monoclonal anti-MHC class I (M0736, DAKO, 1:500); mouse monoclonal anti-MHC class II (M0775, DAKO, 1:200); mouse monoclonal anti-c5b-9 (MO777, DAKO, 1:50); mouse monoclonal anti-CD 31 (MO823, DAKO, 1:500); rabbit polyclonal anti-MxA2 (sc-50509, Santa Cruz, 1:100); mouse monoclonal anti-VEGF (sc 7269, Santa Cruz, 1:100); mouse monoclonal anti-Myotilin (RS034, Novocastra, 1:50); mouse monoclonal anti-MHCD (RNMy2/9D2, Novocastra, 1:20); mouse monoclonal anti-CD56 (18-0152, Invitrogen); mouse monoclonal anti-CD68 (M0814, DAKO, 1:100); mouse monoclonal anti-CD20 (M0755, DAKO, 1:100); mouse monoclonal anti-CD8 (M7103, DAKO, 1:500); and rabbit- monoclonal anti- CD3 (RM-9107-S0, Medac, 1:200) (Supplementary Table S1). Sections were examined with a Nikon eclipse 80i microscope, equipped with a Nikon digital camera DS-Fi1.

The muscle pathology was scored semi-quantitatively according to a modified consensus ranking scale (VAS) aimed at determining the severity of tissue damage published by Wedderburn and Varsani et al. [47,48], including the following prominent domains: inflammation, vascular, muscle fibers, and connective tissue. Muscle pathology was rated from normal or absent (0) to strongly affected (10) on H&E and Gömöri trichrome stained sections. Additionally, the muscle morphology was rated from normal or absent (0) to strong (3) for perifascicular atrophy, punched-out vacuoles (POV), necrotic muscle fibers, regenerative muscle fiber, oedema, and inflammatory cells using H&E stained sections. ALP staining of fragmented perimysial cells and COX-negative or reduced (pale-blueish) fibers were estimated in standard stains. Antibody-stained sections were rated from physiological expression or absent (0) to strong (3). A reduction in vessel density was rated on anti-CD31 stained sections from normal (0) to strong (3). Three investigators (AS, LR, and WS) were blinded to the specimens during examination. Both AS and WS are experienced with semi-quantitative analysis of muscle sections and have performed similar studies previously [7,49,50,51].

#### 2.2.2. Transmission Electron Microscopy (TEM)

Samples for TEM analysis were available for 13/15 patients. Small samples were taken from open muscle biopsies, fixed in 4% glutaraldehyde/0.4 MPBS, and processed according to standard procedures. For contrast in transmission electron microscopy (TEM), ultrathin sections were treated with 3% lead citrate-3H_2_0 with a Leica EM AC20 (ultrastain kit II) and examined at a Zeiss EM109 TEM, equipped with a sharp eye digital camera. Muscle pathology was scored from normal or absent (0) to strong (3), and myofibrillar disintegration, Z-band alterations, and glycogen deposits were analyzed in longitudinal sections at magnification 12,000×. Mitochondrial pathology and tubuloreticular deposits (TIR) in endothelial cells of endomysial capillaries were analyzed in cross sections at magnification 12,000×. At least 10 endomysial capillaries in each specimen were analyzed. The presence of deposits was rated from 0 to 3. Myonuclear actin inclusions were studied in selected cases with ASyS (P4, P14, P6). At minimum, 200 nuclei were analyzed in each specimen.

### 2.3. Statistical Analysis

To determine whether there were differences among jIIM subgroups DM, ASyS, and OM, with respect to their morphological characteristics (semi-quantitative variables), the Kruskal–Wallis test was performed. Age at biopsy, VAS score, and laboratory parameters were also compared using the Kruskal–Wallis test. All *p*-values were adjusted for multiple testing. R version 4.0.3 was used to perform the analysis.

## 3. Results

### 3.1. Clinical Data

**Figure 1:**
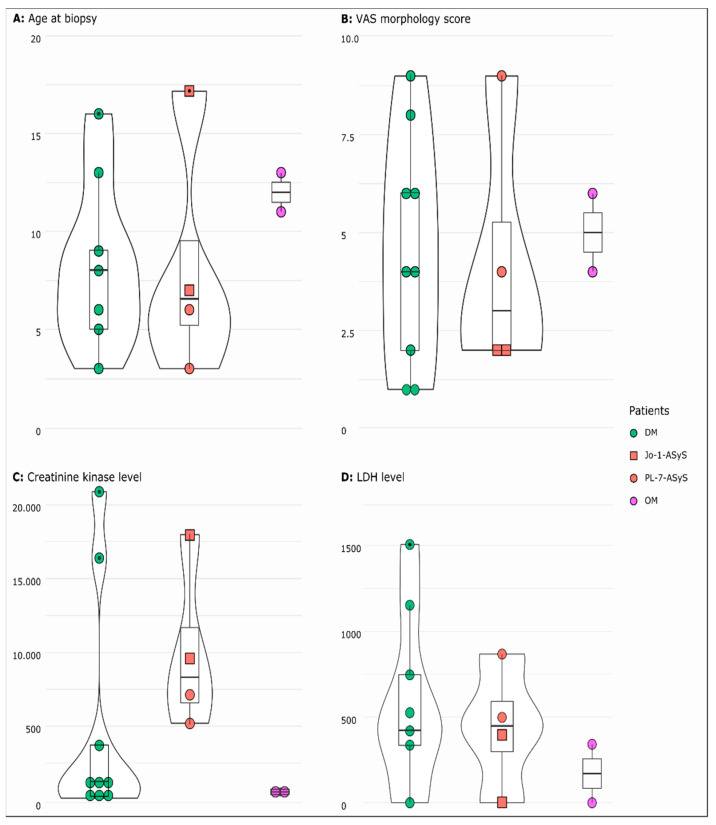
Distribution as violin plot (rotated kermel density curve and boxplot) of age at biopsy (**A**) VAS morphology score (**B**), serum creatine kinase (**C**), and LDH (**D**) levels in jIIM subgroups DM, ASyS (Jo-1, PL-7), and OM. Wide sections represent a higher probability that the patients take the value and small sections represent a lower probability. The box represents the interquartile range, the whiskers minimum (Q1 − 1.5 × IQR) and maximum (Q3 + 1.5 × IQR), and the points show the outliers. For all parameters, the *p*-value did not reach statistical significance. (DM = dermatomyositis; ASyS = antisynthetase syndrome; OM = overlap myositis).

**Figure 2:**
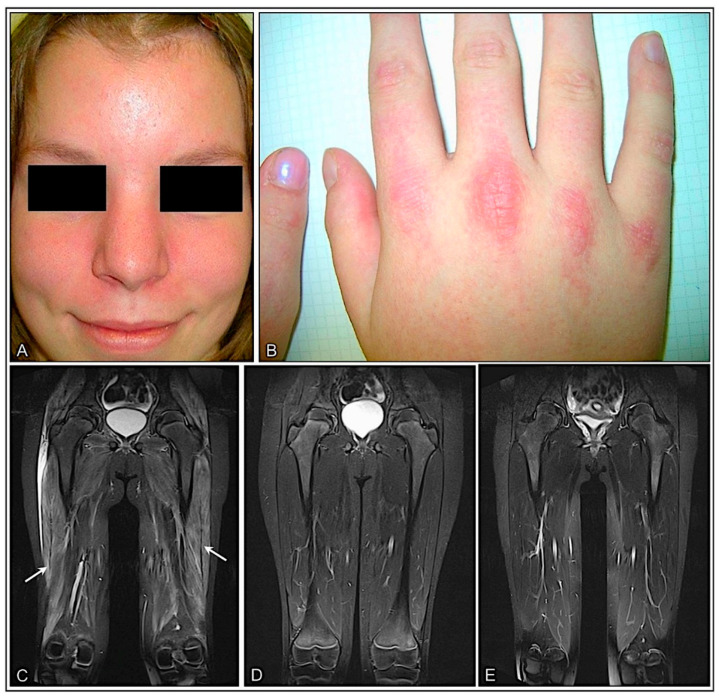
Proximal myopathy and skin lesions are the clinical hallmarks in patients with jIIM. Mild facial erythema (**A**) and discrete erythematous papules overlying the metacarpal joints (Gottron’s papules) (**B**) in a 16-year-old girl with DM (P5). Coronal MR images in a 10-year-old patient with anti-NXP2 antibody-associated DM (P4) showing mild contrast enhancement in T1-weighted images and diffuse oedema of the thigh muscles at first presentation (arrows) (**C**) resolving completely with treatment after 6 (**D**) and 12 months (**E**).

Fifteen patients were included in the study. Median age at biopsy was 8 (range 3–17) years, of whom of 73% were females (Figure 1A). Duration of disease was subacute (>14 days) in the majority of patients. Dermatological manifestation (100%), muscle weakness (93%), and myalgia (73%) were the key diagnostic elements. Muscle weakness was particularly present in the proximal lower limbs with difficulties in climbing stairs or getting up from the chair. Fatigue (47%) and mood swing with sadness (33%) were reported. Four patients (27%) developed calcinosis. Extramuscular manifestation with pulmonary (20%) and cardiac involvement (27%) occurred (Table 1 and Table S2). Specific EMG signs, characteristic of dermato-polymyositis, such as abundant abnormal spontaneous activity (e.g., small positive waves firing at slow rates, defibrillation potentials, myotonic discharges), in conjunction with short polyphasic muscle unit potentials of low amplitude, were detected in 72%. Rather unspecific alterations, such as rare spontaneous activity and a mixture of some polyphasic low-amplitude and normal muscle unit potentials, were present in 27%. Typical muscle MRI findings of dermato-polymyositis, such as marked T2 hyperintensity in the thigh muscles with edema on T2-fat suppressed sequences and marked contrast enhancement on post-gadolinium sequences, were detected in 73%. Characteristic ultrasound abnormalities, such as diffusely increased echogenicity and blurred fibrillar echotexture of the muscle, as well as thickened fascia, were seen in 67% (Table 1 and Table S4). Figure 2 shows characteristic skin involvement (A, B) and MRI findings (C-E) of patients with IIM.

Out of 12 children, 6 (50%) had autoantibodies, with anti-NXP-2 (2/12), anti-Jo1 (2/12), and anti-PL-7 (2/12). In two patients, anti-nRNP/Sm antibodies were detected. Anti-nuclear antibodies (ANA) were positive in 69%. Elevated serum CK and LDH levels were the most prominent laboratory features (Figure 1C,D). ASyS patients generally showed higher CK levels compared to the DM and OM groups but did not reach statistical significance (*p* > 0.05) (Figure 1C,D). Extramuscular symptoms did not occur in anti-PL-7-ASyS and OM. Comorbidities were higher in anti-PL-7-ASyS and OM. Accompanying symptoms, such as fatigue, was present in all subgroups. Myalgia was not noted in the two patients with anti-Jo1-ASyS (Supplemementary Table S3). Muscle biopsies had been taken before start of therapy in 13/15 patients. Only P13 and P4 received pulse steroid therapy shortly before biopsy.

### 3.2. Muscle Pathology

#### 3.2.1. Muscle Pathology Score and Inflammatory Cell Invasion Are Highly Variable in All IIM Subtypes

Seven non-diseased control muscle biopsies showed no significant pathology and served as a morphological baseline for standard enzymatic and immunohistochemical studies and VAS score (Supplementary Table S6).

**Figure 3:**
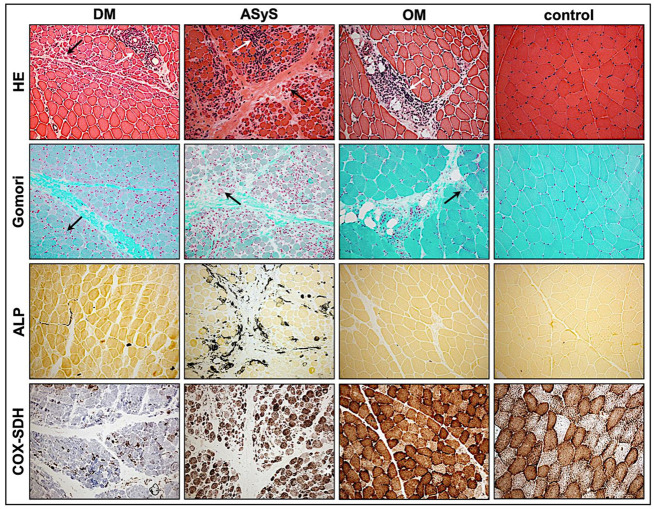
Representative staining of standard stained sections of jIIM from patients with DM (P4), anti-Jo1-ASyS (P14), OM (P3), and control. In H&E-stained sections, perifascicular atrophy (black arrows) is prominent in biopsies from patients with DM and anti-Jo1-ASyS. In OM, atrophic fibers are distributed throughout the section. Inflammatory cell infiltrates (white arrows) are mainly located perifascicularly in DM, perifascicularly in anti-Jo1-ASyS, and perimysial in OM sections. In Gömori trichrome, muscle fibers show a strong alteration of myofibrillar structures in DM and anti-Jo1-ASyS (arrows) but not in OM. ALP is highly upregulated (black) in the perimysium of anti-Jo1-ASyS and shows some subtle upregulation in DM but not in OM. COX-negative fibers appear blue in the COX–SDH staining with an exclusively high number of COX-deficient fibers in the DM sections (magnification 20×). (DM = dermatomyositis; ASyS = antisynthetase syndrome; OM = overlap myositis).

**Figure 4:**
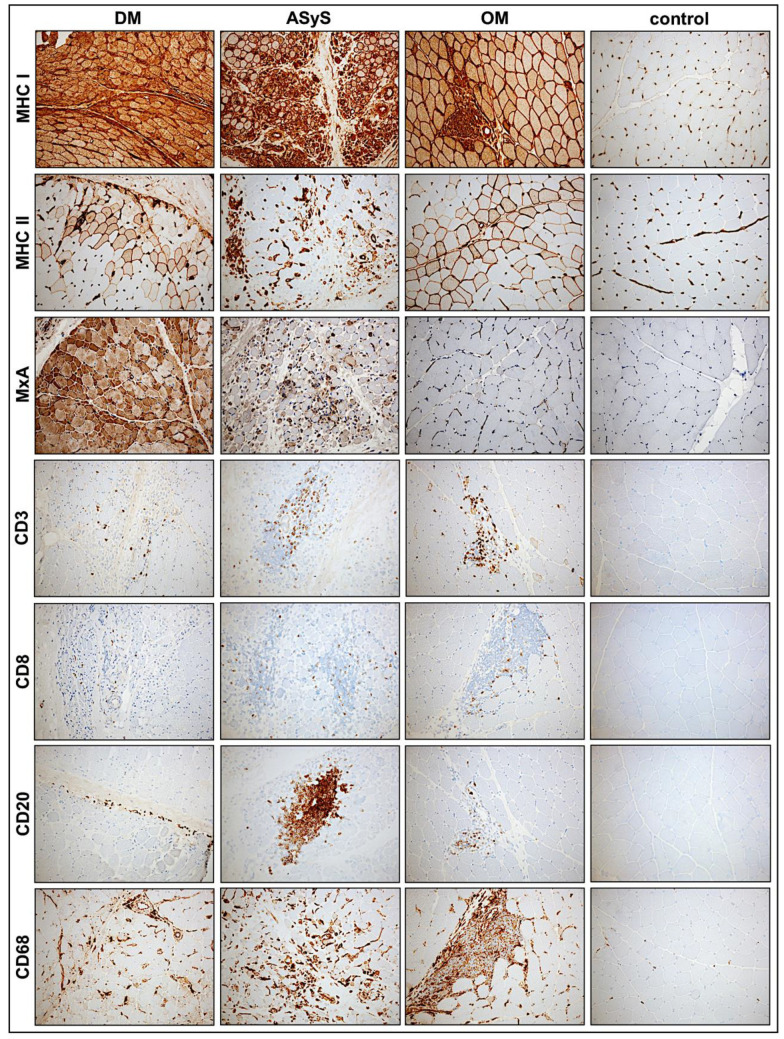
Representative staining of immunohistochemical features of biopsies from patients with DM (P4), anti-Jo1-ASyS (P14), OM (P3), and controls. The upregulation of MHC class 1 on muscle fibers is ubiquitously strong in all IIM subtypes, whereas MHC class II is mainly expressed at perifascicular muscle fibers. Upregulation of MxA is the highest in DM but also present in anti-Jo1-ASyS. Inflammatory cells occur in all subtypes, mainly T-lymphocytes (CD3, CD8). B-lymphocytes (CD20) are prominent in Jo-1-ASyS. Macrophages (CD68) are prominent in all subtypes (magnification 20×). (DM = dermatomyositis; ASyS = antisynthetase syndrome; OM = overlap myositis).

The muscle pathology (severity) score VAS showed a high heterogeneity in all subgroups with the highest score of 9 present in an anti-NXP-2-DM patient and in an anti-Jo-1-ASyS patient. There was no significant difference regarding the overall pathological severity score between DM, ASyS, and OM (*p* = 0.8979) (Figure 1B). Perifascicular atrophy and punched-out vacuoles (POV) were mainly seen in DM and anti-Jo-1-ASyS, whereas necrotic fibers, oedema, and regeneration were present in all IIMs. In two cases with OM, the VAS score was high (4–6) with many necrotic and regenerating fibers. P13, with the diagnosis of anti-NXP-2-DM, showed a weak pathology morphology in all sections, consistent with a mild clinical phenotype and normal CK levels (Figure 3, Table A1). Inflammatory cells were present in all IIM muscle specimens, presenting as mainly T-cells (CD3, CD8) and macrophages (CD68). B-cells (CD20) were present only in few cases of all subgroups (Figure 4, Table A2). 

#### 3.2.2. COX Deficient Fibers Are a Striking Pathology in DM Biopsies

Only in DM skeletal muscle biopsies, perifascicular COX-deficient fibers were detectable, albeit with a high variability from 0–3. COX-deficient fibers were absent in ASyS or OM cases (Table A1, Figure 3).

#### 3.2.3. Perimysial Alkaline Phosphatase (ALP) Positivity Is Specific for Anti-Jo-1–ASyS

Alkaline phosphatase (ALP) positivity of fragmented perimysial tissue is a specific finding to discriminate ASyS cases from other IIM subtypes [41,52]. Strong perimysial staining with ALP was only present in two cases of anti-Jo-1-ASyS, but not in PL-7-ASyS or other IIM cases consistent with findings in adult patients [39] (Table A1, Figure 3). 

#### 3.2.4. Sarcolemmal Upregulation of MHC Class I, MHC Class II and Sarcolemmal Complement Deposits

**Figure 5:**
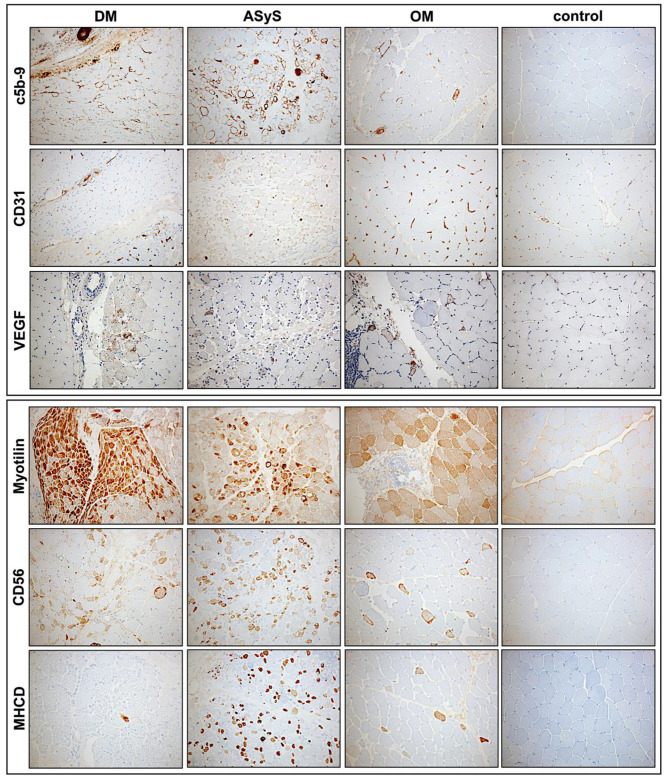
Representative stains of immunohistochemical features of biopsies from patients with DM (P4), anti-Jo1-ASyS (P14), OM (P3) and control. In DM, complement deposits (C5b-9) on endomysial capillaries are strong at the perifascicular region but can also be present in anti-Jo1-ASyS. Strong complement deposits on myofibers are present in anti-Jo1-ASyS in a perifascicular pattern. Few complement deposits are present on scattered fibers in OM. CD31^+^ endomysial capillaries are reduced in DM and anti-Jo1-ASyS. Focal reduction in CD31^+^ vessels is also present in OM. Upregulation of vascular endothelial growth factor (VEGF) is mainly seen in DM but can also occur in anti-Jo1-ASyS and OM. Myotilin staining indicates a disorganization of sarcomeric structure. Strong perifascicular sarcomeric disruption is present in DM and less strong in anti-Jo1-ASyS. With antibodies against CD56 and MHCD, a strong regenerative capacity is seen in anti-Jo1-ASyS (magnification 20×). (DM = dermatomyositis; ASyS = antisynthetase syndrome; OM = overlap myositis).

MHC class I was strongly upregulated on the muscle fiber sarcolemma in almost every biopsy (14/15) of jIIM, confirming the diagnosis of myositis [53]. MHC1 showed a diffuse upregulation pattern in most cases with a perifascicular gradient of varying degree in the majority of DM and anti-Jo-1-ASyS. No perifascicular MHC class I upregulation was seen in OM and PL7-ASyS. MHC class II sarcolemmal upregulation was present in the majority of the cases but less strong with a scattered distribution in most cases, in line with previously shown results [53]. In two DM cases (P2, P12) with strong pathology, a perifascicular staining pattern of MHC class II was more pronounced. Sarcolemmal deposits (C5b-9) on muscle fibers were present in 3/9 DM, 2/4 ASyS, and 1/2 OM with the strongest expression in an anti-Jo-1-ASyS, predominately in the perifascicular region (Table A2, Figure 4 and Figure 5).

#### 3.2.5. Vascular Pathology and Upregulation of Proangiogenic Factor VEGF

Vascular pathology might contribute to muscle damage in jIIM. Strong complement deposits (C5b-9) were present on endomysial capillaries in DM (7/9) and anti-Jo-1-ASyS (2/2), whereas no capillary deposits were detected in anti-PL-7-ASyS and OM (Table A2, Figure 5). A reduction in CD31+ endomysial capillaries was more prominent in DM and anti-Jo1-ASyS compared to anti-PL7-ASyS and OM. The proangiogenic factor VEGF was upregulated with perifascicular distribution in DM and anti-Jo-1-ASyS cases. Moderate rarefication of CD31+ capillaries, and VEGF upregulation were also present in OM (Table A2, Figure 5).

#### 3.2.6. Ultrastructural Pathology

**Figure 6:**
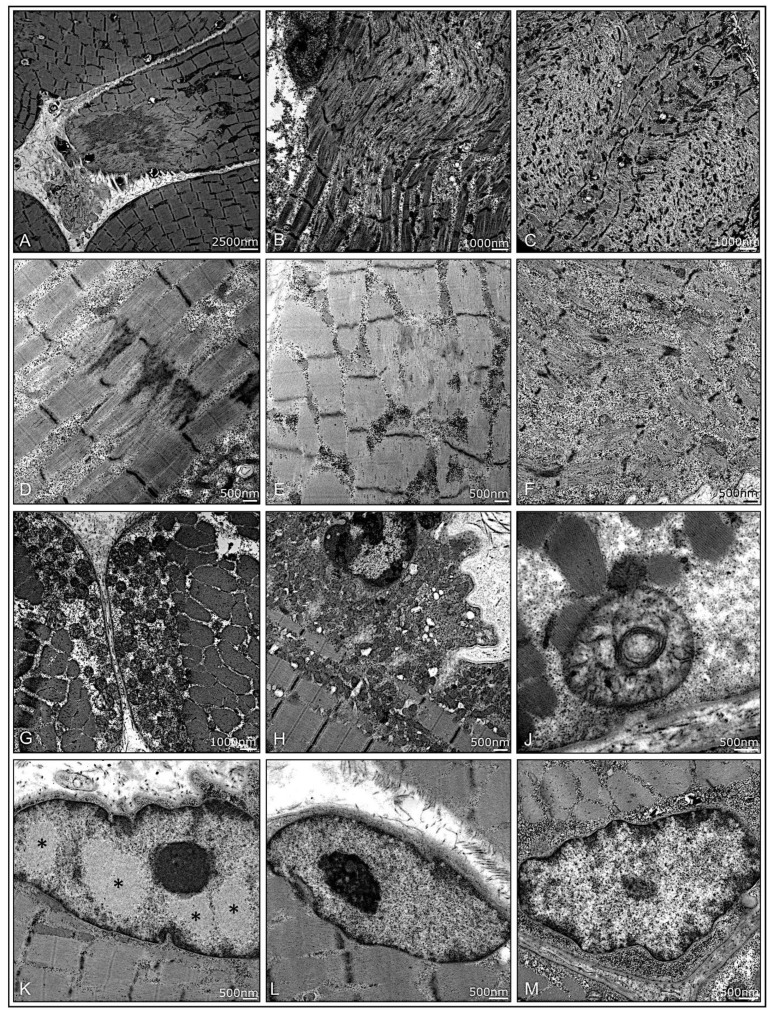
Ultrastructural pathology in muscle biopsies from patients with jIIM. A high variability of disruption of myofibrillar architecture can be present in all subtypes. Focal, core-like alterations of the sarcomeric architecture in DM (P10) (**A**), P4 (**B**), and P12 (**C**). Z-band alterations with Z-band streaming in P15 (**D**) and dissolving Z-bands in anti-Jo1-ASyS (P14) (**E**) and focal glycogen deposits in OM (P7) (**F**). Moderate mitochondrial pathology with some mitochondrial subsarcolemmal aggregation in PL-7-ASyS (P9) (**G**) and DM (P2) (**H**). Few mitochondria with increased variability of diameter and altered cristae structure in P9 (**J**). Myonuclei with characteristic filamentous nuclear inclusions were only found in anti-Jo1-ASys (P14); note the different patterns of euchromatin, heterochromatin, and the nucleolus, as compared to the nuclear inclusions (*) (**K**). No inclusions were found in PL-7-ASyS (P6, P9) (**L**,**M**). (DM = dermatomyositis; ASyS = antisynthetase syndrome; OM = overlap myositis).

Analyzing the muscle fiber at ultrastructural level shows disruption of myofibrillar architecture with a high variability in all subtypes (Table A3, Figure 6A–F). Mitochondria did show only mild pathology in few samples of IIM with subsarcolemmal aggregation, increased size in diameter (polymorphism), and disruption of cristae structure without any distinct differences between the subgroups (Table A3, Figure 6G–J). Analyses of three cases of ASyS revealed characteristic filamentous nuclear inclusions in 3/410 examined myonuclei, only in anti-Jo-1 ASyS (P14). Due to a section thickness of approximately 200 nm, the substructure of inclusion appeared slightly more homogenous, as compared to 60–100-nm sections. No nuclear inclusion was found in ASyS-PL-7 (P6, P9) (Table A3, Figure 6K–M).

#### 3.2.7. IFN 1 Surrogate Marker MxA Upregulation Correlates with Endothelial Inclusions

**Figure 7:**
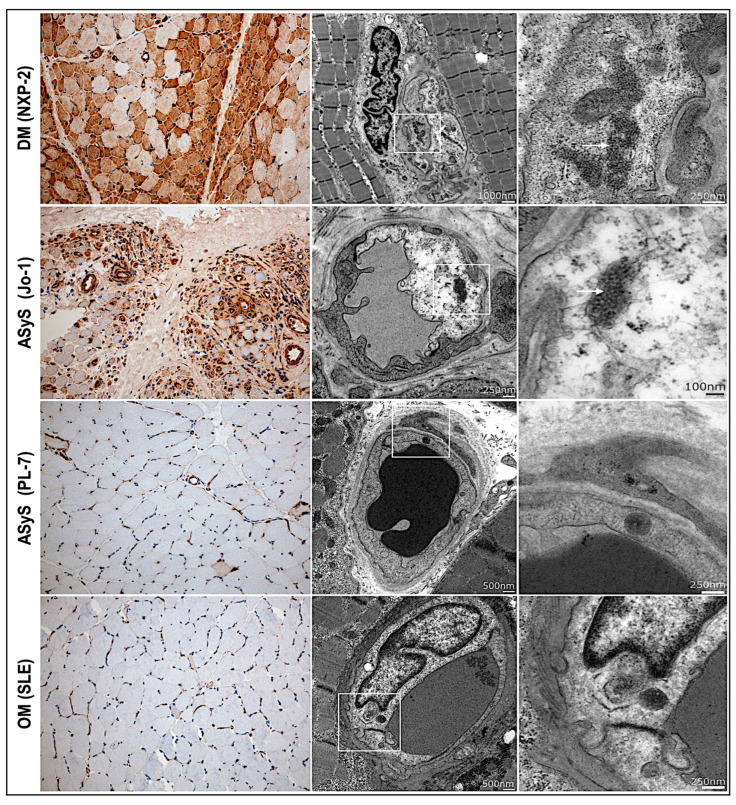
MxA upregulation and tubuloreticular inclusions in muscle biopsies from patients with IIM. Strong perifascicular upregulation of interferon surrogate marker MxA in DM (P4) and anti-Jo-1 ASyS (P14) associated with tubuloreticular inclusions (TIR) in the endothelial cells of small vessels. Weak MxA upregulation on scattered myofibers with nonspecific osmiophilic inclusions in anti-PL7 ASyS (P6) and OM (P3) (magnification 20×) (DM = dermatomyositis; ASyS = antisynthetase syndrome; OM = overlap myositis).

Myxovirus resistance protein A (MxA) is a type I-IFN-induced protein and serves as a diagnostic tool to discriminate DM from other IIM subtypes in juvenile and adults, which are at least equivalent in diagnostic performance compared to MHC class I staining [54]. In our study, MxA staining showed strong upregulation on the sarcolemma of myofibers and vessels with a perifascicular predominance distribution in 8/9 patients with DM. The MxA expression correlated with the presence of endothelial tubuloreticular inclusions (TRIs) at TEM. In two patients with Jo-1-ASyS sarcoplasmic and vessel staining by MxA, an antibody was prominent, similar to those of the DM cases. TRIs were also frequent in one of the Jo-1-ASyS, and the only case of Jo-1-AsyS was available for TEM. No significant perifascicular MxA upregulation or endothelial TRI were found in patients with anti-PL-7–ASyS (n = 2), OM (n = 2), or in the controls (n = 7). Only few scattered fibers with weak MxA upregulation were detected in P6 (anti-PL-7-ASyS) and P3 (OM) (Table A3, Figure 7).

#### 3.2.8. Moderate Activation of Autophagy in jIIM

**Figure 8:**
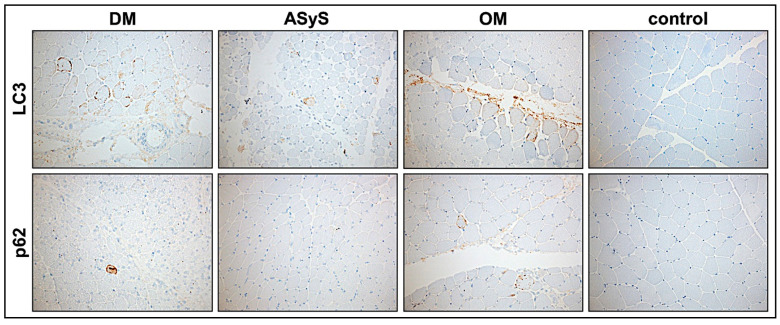
Expression of autopathic markers LC3 and p62 in jIIM muscle biopsies of patients with DM (P4), ASyS (P14), OM (P3), and control. Few muscle fibers expressing LC3 and p62 are present in DM (P4), ASyS (P14), and OM (P3), indicating a moderate upregulation of autophagy (magnification 20×). (DM = dermatomyositis; ASyS = antisynthetase syndrome; OM = overlap myositis).

We analyzed the expression of the prototypic autophagy-related markers, p62 and LC3, in muscle biopsies from jIIM patients compared to controls. Few LC3 or p62-positive inclusions (rating from 0.5-1) were shown in 5/15 biopsies. In sections with a higher score (P12-score 8, P4-score 9, P14-score 9), LC3 expression was more pronounced. Additionally, in these cases, the muscle fiber regeneration (CD56, MHCD) was higher (Table A3, Figure 8).

#### 3.2.9. Common Staining Pattern and Discriminative Key Findings in jIIM Subtypes

**Figure 9:**
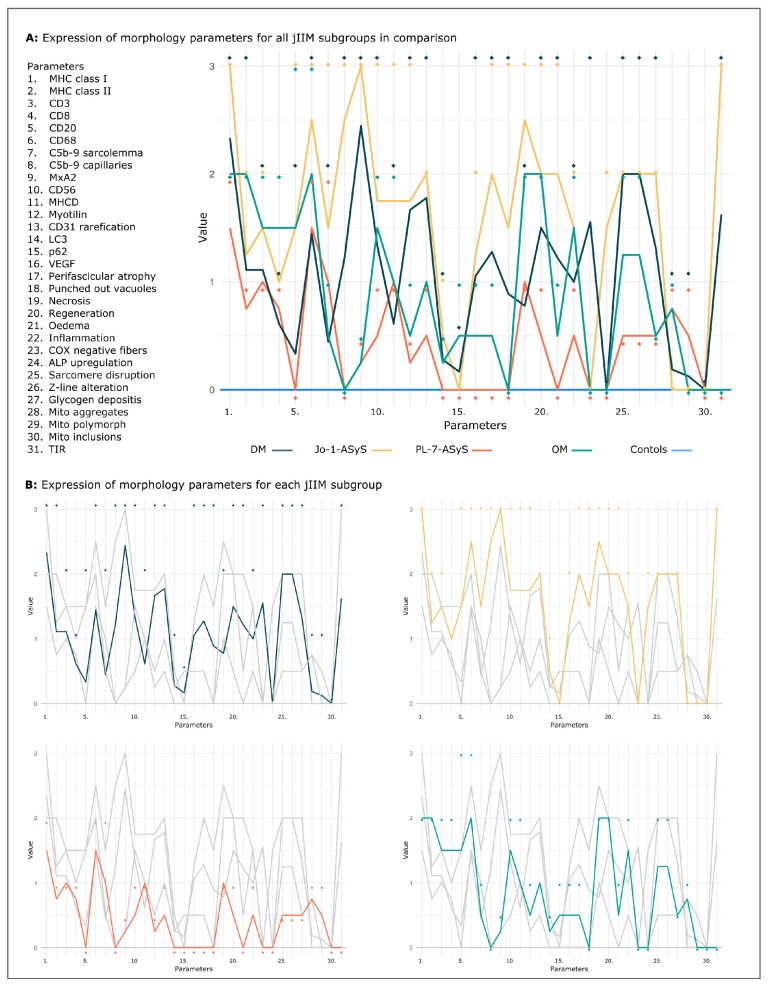
Visual exploration of the values. Common and discriminative morphological patterns in jIIM subgroups DM, ASyS, OM, and controls for 31 parameters, shown as mean (coloured lines) and maximum values (rhombus) per subgroup as an overview (**A**) and separated by subgroups (**B**). The minimum values per subgroup representing weak morphological characteristics are not shown. Healthy control (blue line) shows the normal pattern. Note the visible similar trend of the pattern of DM and Jo-1- ASyS compared to that in PL-7- ASyS and OM. For all parameters, the *p*-value did not reach statistical significance. (DM = dermatomyositis; ASyS = antisynthetase syndrome; OM = overlap myositis, TIR = tubuloreticular inclusions).

Comparing the detailed analysis of thirty-one parameters shows common and distinct features, which are helpful to discriminate the different jIIM subtypes from each other. Muscle biopsies from controls showed a normal staining serving as a baseline. Common features in all jIIM muscle biopsies included the upregulation of MHC class I, lympho-monocyte cell infiltrates, scattered muscle fiber necrosis, and myofiber regeneration. Mitochondrial pathology with COX-negative fibers were exclusively present in DM. Anti-Jo-1-AsyS, but not PL-7-ASyS, shared some common features with DM, including perifascicular MxA upregulation, rarefication of CD31^+^ vessels and capillary C5b-9 deposits with endothelial inclusions (TIR), and punched-out vacuoles. Staining of the fragmented perimysium by ALP was present only in Jo-1-ASyS cases. Additional sarcolemmal C5b-9 deposits were more striking in Jo-1-ASyS. OM biopsies showed common morphological features of myositis, including with more scattered distribution compared to DM and anti-Jo-1-AsyS. Perifascicular atrophy can occur in OM, but without perifascicular upregulation of MHC class 1 or MxA (Figure 9A,B).

## 4. Discussion

The aim of this study was to analyze childhood myositis subtypes by using a comprehensive panel of stains that are widely available and recommended for diagnostic purposes in inflammatory myopathies [55]. This was carried out explicitly in a single tertiary center to avoid any confounders (including technical ones), and to gain full access to all additional data of the patients. Here, we describe that there are obvious and well-discernible morphological differences between DM and ASyS-associated myositis and overlap myositis in children. Those differences are well in line with current hypotheses about the different pathogeneses and the immune mechanisms involved in these entities. As we are approaching the era of targeted and individualized therapies, tailored according to the specific etiopathogenesis of diseases, it is of outmost importance to have a fine-tuned diagnostic repertoire that reflects immune pathogenesis in jIIM.

The juvenile patients enclosed in our study showed clinical signs of an IIM with the leading symptoms of subacute manifestation of proximal weakness and skin symptoms. The weakness was prevailing in the proximal lower legs with difficulties in climbing stairs. The diagnosis of jIIM was verified by MRI, EMG, and/or muscle ultrasound and laboratory data with an increased CK in the majority of patients. The distribution of age and gender with a higher proportion of affected females was in line with that of other studies [10,22]. Interestingly, in 33% of our young patients, sadness and mood swing were overt, which juxtaposes clinically reported signs of adult patients with IIM. Calcinosis, a skin symptom in jDM and uncommon in adult DM, was present in one third of our patients [24].

Compared to adults, the sub-classification of IIM in children is less well defined [3,4,6,54,56]. In our cohort, the majority of cases were diagnosed as DM, followed by ASyS and OM. MSA were analyzed in the majority of the patients, and in 50% of the patients, antibodies were detected, showing anti-NXP-2, anti-Jo1, and anti-PL-7 antibodies.

For morphological classification of juvenile IIM, only few studies were performed so far but without including all IIM subtypes and without using a specific and comprehensive myopathological repertoire of stains [33]. A common pathology in all of our juvenile IIM cases was the upregulation of MHC class 1 and MHC class 2, inflammation, oedema, necrotic fibers, and regeneration. Lympho-monocyte cell infiltrates were present in all subtypes. Compared to other studies, B-lymphocytes were not the predominant cell type in our DM cohort, but also occurred in Jo-1-ASyS and OM [57]. These analyses are mandatory to delineate IIM from hereditary myopathies. However, the staining alone does not discriminate between the distinct subtypes. Using detailed morphological analysis of thirtyone parameters showed a number of differences in staining patterns among the IIM subtypes.

Chronic disturbance in endothelial cell homeostasis, leading to a vasculopathy, is considered a major contributor to jDM, and markers of endothelial injury are increased in active jDM [58,59,60]. Severe vascular pathology predicts a chronic disease course and suggests poor prognostic factor in the outcome of jDM [60]. In muscle biopsies, a vasculopathy characterized by loss of endomysial vessels and hypoxia with upregulation of VEGF predominantly in perifascicular regions has been described with a strong heterogeneity in jDM patients [51].

Our data confirm the involvement of vascular pathology in jDM. A strong vasculopathy in jDM is associated with a reduced capillary network, capillary complement deposits, tubuloreticular endothelial inclusions, and upregulation of hypoxic marker (such as VEGF and strong muscle fiber pathology with disruption of the myofibrillar structure and hypoxia-driven pathology).

Vascular pathology was also present in anti-Jo-1-ASyS, leading to the hypothesis of a common pathway in pathogenesis compared to DM [39]. Vascular rarefication and VEGF upregulation also occurred in OM cases. These findings lead to the hypothesis that vascular involvement might play a role in pathogenesis, in inflammatory myopathies other than DM.

Microvascular membrane attack complex deposits in dermatomyositis might result from activation of the classical complement pathway triggered by direct binding of C1q to injured endothelial cells [61]. In our cohort, capillary complement deposits (C5b-9) were present in patients with jDM and anti-Jo-1-ASyS. Sarcolemmal complement deposits were present with strong perifascicular expression in anti-Jo1-ASyS. This underlines the hypothesis that, in ASyS, complement decoration of myofibers is involved in interferon (IFN)γ-mediated myofiber damage in that specific area.

IFN-induced reactive oxygen species and mitochondrial damage contribute to muscle impairment and inflammation maintenance in dermatomyositis [62]. COX-deficient fibers are present in adult DM muscle samples with a variability different in MSA subgroups [56]. In our jIIM patients, COX-deficient fibers were exclusively present in DM muscles; thus, we can attach more importance to this staining for differentiating between certain subgroups. However, manifest mitochondrial ultrastructural alterations with formation of paracrystalline inclusions (as in monogenic mitochondriopathies or in IBM) were not seen.

Type I interferon (IFN) upregulation plays a key role in jIIM and inhibitory regulators of IFN, such as ISG15, as well as discriminated patients with DM from those with OM and inversely correlated with the severity of muscle pathology and positively with the clinical outcome [30]. The IFN surrogate marker, MxA, has a high specificity and sensitivity in DM cases and is recommended for muscle biopsy diagnosis by international consensus [54,63]. A perifascicular upregulation of MxA was seen in all of our jDM biopsies, with the exception of one case with an overall low pathology and sparse clinical symptoms. Strong MxA expression on muscle fibers and vessels was also present in one anti-Jo-1-ASyS case, but only weak or absent in anti-PL-7-ASyS. These data are in line with other studies, showing that MxA is expressed in a subset of AsyS cases. This suggests the possibility of common or overlapping pathological (type I and/or type II) interferon-related pathways in DM and ASyS, especially associated with anti-Jo1 antibodies [64]. This issue clearly warrants further detailed studies in the future. Endothelial inclusions (TIR) were also detected in the anti-Jo-1 ASyS, which is mainly a diagnostic criterion for DM, confirming this hypothesis.

In antisynthetase syndrome, our study highlighted the specific morphological features with perifascicular atrophy. As expected, the ALP staining pattern was strong in anti-Jo-1-ASyS and was not altered in other subgroups; thus, high sensitivity and specificity helps to distinguish ASyS from other myopathies, showing that ALP is a helpful diagnostic marker [39,52]. However, normal ALP staining does not exclude ASyS, but it is unlikely that it renders anti-Jo1-ASyS. The presence of filamentous nuclear inclusions are hallmarks of adult ASyS [41]. Only the specimen of an anti-Jo1-ASyS patient showed characteristic nuclear inclusions, whereas no nuclear inclusions were found in anti-PL-7-ASyS. From the data of our study, anti-Jo-1 and anti-PL-7-ASyS might have different pathological characteristics and should be further analyzed in larger cohorts.

In juvenile OM, the muscle pathology is not analyzed in detail so far. In our cohort, muscle pathology showed common features of IIM with MHC class I upregulation, inflammation, and oedema with a moderate pathology score. Tubuloreticular inclusions can occur in OM, especially in lupus erythematosus, but were not present in our cases [65,66]. MxA upregulation was weak on scattered fibers, but was not in a perifascicular distribution in OM, consistent with the upregulation of type I IFN in childhood SLE [18,67].

Autophagy is important for regulating homeostasis in cells. In skeletal and cardiac muscles, due to tension-induced force, misfolded proteins and damaged organelles are recycled by autophagic processes [68]. P62^+^ inclusion in myofibers are a nonspecific process following muscle injury, including myopathy and neurogenic atrophy, and is more prevalent in biopsies with more severe muscle damage [69]. In IIM, dysregulated or enhanced autophagy is described in IMNM and sIBM with distinct patterns of p62 inclusions and may play a role in the disease pathology [70]. In our study, the autophagic molecules, LC3 and p62, were visible only in few biopsies of DM, and anti-Jo-1-ASyS cases all showed higher scores of autophagy-related pathomorphology. This finding is consistent with an increased autophagy in more strongly affected muscle biopsies and awaits further detailed analysis in comparison to the adult situation.

For adults, a muscle biopsy to confirm the clinical diagnosis of myositis and characterize the myositis subtype is recommended by the German Neurological society, since a precise diagnosis is crucial for the therapy regime. Our data suggest that certain subtypes of myositis also exist in juvenile patients. Because these subtypes are less well defined compared to adults, and since myositis specific antibodies are false positive or missing in about 20% of children, we recommend a muscle biopsy in all juvenile patients with clinical symptoms of myositis. A small biopsy (0.5 cm in diameter) is adequate to perform a large set of staining and electron microscopy. Additionally, studies of larger cohorts are necessary to improve understanding on the underlying aetiopathology of juvenile myositis and therapy options.

## 5. Limitations

This study has some limitations. First, the clinical data from the patients were collected retrospectively. Therefore, MSA were not available for every patient in our cohort. Second, this is a single-center study which has certain advantages; however, the cohort of patients with such rare diseases is small, especially since the group of OM was diagnosed only in two patients with SLE. Therefore, the small sample size reduces the statistical power and the ability to formally identify significant effects in the cohort. Nevertheless, the described and visualized morphological and morphometric patterns of the subgroups provided very useful trajectories to define diagnostic and classification criteria.

## 6. Conclusions

Our study demonstrates the need of detailed and distinct morphological characterization of muscle pathology in juvenile myositis, with respect to precise prognostic and therapeutic decisions in jIIM, especially in children with negative or absent serological findings.

## Figures and Tables

**Table 1:** Summary of demographic data of patients with jIIM.

Patients with jIIM	
Number of patients	15
Age at biopsy (median)	8 years
range	(3–17 years)
female	11 (73%)
**Skeletal muscle symptoms**	**14 (93%)**
Myalgia	11 (73%)
Exercise induced myalgia	6 (40%)
Proximal weakness	14 (93%)
**Skin symptoms**	**15/15 (100%)**
Dry skin	6 (40%)
Exanthema	3 (20%)
Erythema	4 (27%)
Butterfly rash	5 (33%)
Redness, livid coloration	8 (53%)
Raynaud’s phenomenon	1 (7%)
Gottron’s papules	8 (53%)
Nail fold changes	1 (7%)
Calcinosis	4 (27%)
**Accompanying symptoms**	**15/15 (100%)**
Fever	3 (20%)
Difficulty swallowing	1 (7%)
Morning stiffness	1 (7%)
Arthritis	1 (7%)
Weight gain	2 (13%)
Weight loss	1 (7%)
Night sweat	1 (7%)
Oedema	3 (20%)
Lymphadenopathy	1 (7%)
Fatigue, concentration difficulties	7 (47%)
Loss of appetite/rejection to drink	1 (7%)
Sadness/mood slump/mood swings	5 (33%)
Social withdrawal	3 (20%)
**Extramuscular symptoms**	**5/15 (33%)**
Pulmonary restriction	2 (13%)
Pneumonia	1 (7%)
Cardial involvement	4 (27%)
**Comorbidities**	**5/15 (33%)**
Cystic fibrosis	1 (7%)
Factor-V-Leiden mutation	1 (7%)
Hypothyroidism	1 (7%)
Steatosis hepatis	1 (7%)
HLA-B27 enthesitis	1 (7%)
**Laboratory results**	**15/15 (100%)**
Creatine kinase (CK) ↑	14 (93%)
Lactatdehydrogenase (LDH) ↑	11 (73%)
GOT ↑	11 (73%)
GPT ↑	7 (47%)
**Serum antibodies**	
**Myositis-specific-antibodies (MSA)**	**6/12 (50%)**
Anti-NXP-2	2 (17%)
Anti-PL-7	2 (17%)
Anti–Jo-1	2 (17%)
**Myositis-associated-antibodies (MAA)**	**2/12 (17%)**
Anti-Ro52	1 (8%)
PM75	1 (8%)
**Antinuclear antibodies (ANA)**	**9/13 (69%)**
**Systemic Lupus erythematodes (SLE)**	
Anti-double-stranded-DNA-antibodies (Anti-dsDNA)	1 (8%)
Smith-Antibodies (Anti-Sm/Sm-AK)	1 (8%)
Anti RNP/Sm-AK	2 (17%)
**MRI skeletal muscle**	**11/15 (73%)**
Normal	2 (18%)
Oedema	6 (54%)
Enhancement of contrast medium/signal alterations	4 (36%)
Compatible with myositis	8 (73%)
**Musculoskeletal Ultrasound**	**9/15 (60%)**
Oedema	3 (33%)
Enhancement of echogenicity/signal alterations	6 (67%)
Compatible with myositis	6 (67%)
**Electromyography (EMG)**	**11/15 (73%)**
Myopathic, compatible with myositis	8 (72%)
Unspecific sign	3 (27%)

## Data Availability

Data are available at supplemental data.

## Supplementary Materials

The following are available online at https://www.mdpi.com/article/10.3390/cells11010109/s1. Supplemental data. Table S1: Antibodies for IHC stains, Table S2: Detailed clinical data of patients with jIIM. Table S3: Laboratory data of patients with jIIM, Table S4: Electromyography, skeletal MRI and muscle ultrasound of patients with jIIM, Table S5: Morphology analysis of muscle biopsies from controls.

## Abbreviations

ANA = antinuclear antibodies; AP = alkaline phosphatase; ASyS = anti-synthetase syndrome; CK = creatine kinase; COX = cytochrome oxidase; DM = dermatomyositis; IIM = idiopathic inflammatory myopathy; MxA = myxovirus resistance protein A; MSA = myositis specific antibodies; MAA = myositis-associated antibodies; MHC class 1 = major histocompatibility factor class 1; OM = overlap myositis; SLE = systemic lupus erythematosus; SDH = succinate dehydrogenase; TEM = Transmission electron microscopy; TRI = tubuloreticular inclusions; VEGF = vascular endothelial growth factor.

## Appendix A

**Table A1:** Scoring of muscle pathology at standard staining.

	Diagnosis	VAS-Score	HE						COX-SDH	ALP
			Perifascicular Atrophy (PA)	Punched-Out-Vacuoles (POV)	Necrosis	Regeneration	Oedema	Inflammation	COX-Negative Fibers	Upregulation
P1	DM	4	1	0	1	2	1	1	2	0
P2	DM	6	3	1	0	3	1	1	3	0
P5	DM	6	1	3	1	1	3	2	3	0
P10	DM	4	0.5	0	1	1	1	0.5	0	0
P11	DM	1	0	0	0	0	0	0.5	0	0
P12	DM	8	3	1	2	3	2	2	3	0
P15	DM	2	0	0	1	0.5	1	0.5	0	0
P4	DM (NXP-2)	9	3	3	1	3	2	1	3	0
P13	DM (NXP-2)	1	0	0	0	0	0	0.5	0	0
P8	ASyS (Jo-1)	4	1	0	2	1	1	1	0	1
P14	ASyS (Jo-1)	9	3	3	3	3	3	2	0	2
P6	ASyS (PL-7)	2	0	0	1	0	0	1	0	0
P9	ASyS (PL-7)	2	0	0	1	1	0	0	0	0
P3	OM (SLE)	4	1	0	2	2	0	1	0	0
P7	OM (SLE)	6	0	0	2	2	1	2	0	0

DM = dermatomyositis; ASyS = antisynthetase syndrome; OM = overlap myositis; SLE = systemic lupus erythematosus.

**Table A2:** Scoring of muscle pathology at IH staining.

	Diagnosis	Inflammation and Immunoactivation						Complement Deposits (c5b-9)		MxA	Regen- Eration		Sarcomere Disruption	Angiogenesis		Autophagy	
		MHC1	MHC2	CD3	CD8	CD20	CD68	Sarco-Lemma	Capil-laries		CD56	MHCd	Myo-Tilin	CD31 Loss	VEGF	LC 3	p62
P1	DM	2	1	1	1	0	1	2	2	3	2	0	1	1	1	0	0
P2	DM	2	1	1	1	0	1	1	0	3	1	1	2	2	2	0	0
P5	DM	3	0.5	2	1	2	3	0	1	3	3	2	3	2	3	0.5	0.5
P10	DM	3	3	2	1	0	1	0	3	2	0	0	2	2	1	0	0
P11	DM	2	0	0.5	0	0	1	0	0.5	2	0	0	0	1	0	0	0
P12	DM	3	2	1	0	0	2	0	0.5	3	2	2	3	3	2	1	0.5
P15	DM	3	0.5	0.5	0.5	0	1	0	2	3	1	0	2	1	0	0	0
P4	DM (NXP-2)	3	2	2	1	1	2	1	2	3	3	0.5	2	3	0.5	1	0.5
P13	DM (NXP-2)	0	0	0	0	0	1	0	0	0	0	0	0	1	0	0	0
P8	ASyS (Jo-1)	3	0.5	1	1	0	2	0	3	3	0.5	0.5	0.5	2	0.5	0	0
P14	ASyS (Jo-1)	3	2	2	1	3	3	3	2	3	3	3	3	2	2	1	0
P6	ASyS (PL-7)	2	0.5	1	1	0	2	2	0	0.5	0	1	0	1	0	0	0
P9	ASyS (PL-7)	1	1	1	0.5	0	1	0	0	0	1	1	0.5	0	0	0	0
P3	OM (SLE)	2	2	2	2	3	3	1	0	0.5	2	2	1	1	1	0.5	1
P7	OM (SLE)	2	2	1	1	0	1	0	0	0	1	0	0	1	0	0	0

DM = dermatomyositis; ASyS = antisynthetase syndrome; OM = overlap myositis; SLE = systemic lupus erythematosus.

**Table A3:** Scoring of muscle pathology at ultrastructural level.

	Diagnosis	Myofibrillar Structure			Mitochondria			Endomysial Capillaries	Nuclear Inclusions
		Sarcomere Structure Disruption	Z-Line-Alteration	Glycogen Deposits	Aggregation	Polymorphism	Inclusions	Tubuloreticular Structures (TIR)	
P1	DM	1	1	2	1	1	0	2 (3/10)	n.a.
P2	DM	3	3	0	0.5	0	0	2 (4/10)	n.a.
P5	DM	n.a.							
P10	DM	2	2	2	0	0	0	1 (2/10)	n.a.
P11	DM	1	1	3	0	0	0	3 (6/10)	n.a.
P12	DM	3	3	0.5	0	0	0	1 (2/10)	n.a.
P15	DM	3	3	2	0	0	0	1 (2/10)	n.a.
P4	DM (NXP-2)	3	3	1	0	0	0	3 (8/10)	n.a.
P13	DM (NXP-2)	0	0	0	0	0	0	0 (0/10)	n.a.
P8	ASyS (Jo-1)	n.a.							
P14	ASyS (Jo-1)	2	2	2	0	0	0	3 (3/5)	3/410
P6	ASyS (PL-7)	0.5	0.5	0.5	0.5	0	0	0 (0/10)	0/205
P9	ASyS (PL-7)	0.5	0.5	0.5	1	1	0	0 (0/10)	0/210
P3	OM (SLE)	0.5	0.5	0.5	0.5	0	0	0 (0/10)	n.a.
P7	OM (SLE)	2	2	0.5	1	0	0	0 (0/10)	n.a.

n.a = not analyzed; DM = dermatomyositis; ASyS = antisynthetase syndrome; OM = overlap myositis; SLE = systemic lupus erythematodes.
