# Supplementary material for: Morphological Characteristics of Idiopathic Inflammatory Myopathies in Juvenile Patients
Source: Cells. 2021 Dec 30;11(1):109. doi: 10.3390/cells11010109 (PMC8750180; doi:10.3390/cells11010109)
Supplement: Supplementary file 1 [file cells-11-00109-s001.zip › Supplemental Table S1 Antibodies for IHC.pdf]

Supplementary Table S1: Antibodies for immunohistochemistry.

	Primary Antibody	Company	Dilution	Target Structure/ Relevance
MHC class I HLA-ABC Antigen	Monoclonal mouse; clone W6/32; code M0736	DAKO	1:500	Major histocompatibilty complex class I
MHC class II HLA-DP, DQ, DR Antigen	Monoclonal mouse; clone CR3/43; code M0775	DAKO	1:200	Major histocompatibilty complex class II
C5b-9	Monoclonal mouse; clone aE11, code M0777	DAKO	1:50	Complement deposits
CD 3	Monoclonal rabbit; clone SP7; RM-9107-S0	Medac – Neomarkers	1:200	T-cell receptor
CD 3	Monoclonal rabbit; clone SP7, RBK024	Zytomed	1:200	T-cell receptor
CD 8	Monoclonal mouse; clone C8/144B, code M7103	DAKO	1:500	Cytotoxic T-cells
CD 20	Monoclonal mouse; clone L26; code M0755	DAKO	1:100	B-cells
CD 68	Monoclonal mouse; clone KP1; code M0814	DAKO	1:200	Macrophages
CD 56	Monoclonal mouse; clone 123C3, code 18-0152	Invitrogen	1:300	muscle fiber regeneration
MHCD	Monoclonal mouse; clone RNMy2/9D2; NCL-MHCd	Novocastra (Leica)	1:20	muscle fiber regeneration
myotilin	Monoclonal mouse; clone RSO34; NCL-MYOTILIN	Novocastra (Leica)	1:50	Sarcomeric structure
p62	Monoclonal mouse; clone 3/P62 LCK LIGAND; 610832	BD Transduction Laboratories	1:500	Autophagy Sequestosome 1
LC3	Monoclonal mouse; clone 5F10; 0231- 00BIOTIN/LC3 -5f10	Nanotools	1:100	Autophagy Microtubule-associated protein 1A
CD 31	Monoclonal mouse; clone JC70A; code M0823	DAKO	1:500	Endothelial cells

MxA2	Polyclonal rabbit (H-285), sc-50509	Santa Cruz Biotechnology	1:100	Myxovirus Res-istance Protein A
VEGF	Monoclonal mouse (C-1), sc-7269	Santa Cruz Biotechnology	1:100	Vascular endothelial growth factor
