# Supplementary material for: Morphological Characteristics of Idiopathic Inflammatory Myopathies in Juvenile Patients
Source: Cells. 2021 Dec 30;11(1):109. doi: 10.3390/cells11010109 (PMC8750180; doi:10.3390/cells11010109)
Supplement: Supplementary file 1 [file cells-11-00109-s001.zip › Supplemental Table S2 Clinical data of patients with jIIM.pdf]

Supplementary Table S2: Clinical data of patients with Jiim.

	D	G	Age *	Biopsy location	Immuno-suppres-sion	Duration	Myalgia	Proxi-mal weak-ness	Skin symptoms	Accompanying symptoms	Extramuscular symptoms	Comorbi-dities
**P1**	DM	m	9	QFM		subacute 4 weeks	+	+	exanthema on upper arms and thighs	loss of appetite, rejection to drink social withdrawal, weight gain	pulmonary restriction, pericardial effusion	hepatic steatosis
**P2**	DM	f	5	QFM		Chronic, first manifest-ation 2004, relapse 2010	-	+	exanthema/ livid redness on the nose, Gottron's Papules	loss of power, concentration difficulties	-	-
**P3**	OM (SLE)	m	13	QFM		subacute	+	+	dry red lesions on the ankles, knees, and elbows	inguinal lymphadenopathy	-	SLE, hypothyroidism
**P4**	DM (NXP-2)	f	9	QFM	Pulse steroid therapy	subacute 8 weeks	+	+	redness on the root of the nose, calcinosis on the elbow and dry skin	Sadness, social withdrawal	pulmonary restriction	-

**P5**	DM	f	13	QFM		subacute	+	+	dry reddened skin, butterfly rash, erythema on the forehead and the upper arms, Gottron's Papules	difficulty swallowing, sadness	mitral regurgitation	factor-V-Leiden mutation
**P6**	ASyS (PL-7)	f	7	QFM		subacute	-	+	dry lesions on the extensor sides of the elbows, exanthema on the chest and arms, cheek erythema, Gottron's Papules	fever, sadness, mood slump	-	-
**P7**	OM (SLE)	m	11	QFM		chronic over 4 years (refugee family from Syria)	+	+	dry lesions on the extensor sides of the elbows, butterfly rash, Raynaud's phenomenon, Gottron's Papules, micro calcinosis on the forearms and hands	loss of power	-	SLE
**P8**	ASyS (Jo-1)	m	6	QFM		subacute	-	-	reddened forearms and elbows, Gottron's Papules, calcinosis on the trunk, thighs and the extensor sides of the arms	mood swings and slump	-	-

**P9**	ASyS (PL-7)	f	17	QFM		subacute 2,5 months	+	+	Gottron's Papules	fatigue and loss of power	-	Cystic fibrosis (severe exocrine and endocrine pancreatic insufficiency , CF-hepatopathy)
**P10**	DM	f	3	QFM		no information	+	+	red cheeks, Gottron's Papules, nail fold changes	fever, arthritis	obstructive pneumonia	-
**P11**	DM	f	5	QFM		subacute 4 months	+	+	butterfly rash	night sweat, oedema of the upper arms and eye lids, weight gain	-	-
**P12**	DM	f	6	QFM		subacute	+	+	dry lesions on elbows and knees, calcinosis of the left lateral thigh	social withdrawal	Tricuspid insufficiency	-
**P13**	DM (NXP-2)	f	16	DeM	Pulse steroid therapy	subacute	+	+	erythema, reddened joints	morning stiffness, oedema (joints),	-	HLA-B27 enthesitis

										fatigue, loss of power		
**P14**	ASyS (Jo-1)	f	3	QFM		subacute 4-5 weeks	-	+	livid periocular coloration, also redness on the forehead and chin, reddened hands, feet and knees, Gottron's Papules	weight loss, fatigue, loss of power	mitral regurgitation, right ventricular overload	-
**P15**	DM	f	8	QFM		no information	+	+	butterfly rash, reddened hands	fever, oedema (face, abdomen, joints), mood slump, concentration difficulties	-	-

D= diagnosis; G= gender; Age* = age of biopsy; QFM=Quadriceps femoris muscle; DeM= Deltoid muscle; DM= Dermatomyositis, ASyS= Antisynthetase Syndrome, OM= Overlap-Myositis; SLE= Systemic lupus erythematosus
