# Supplementary material for: Morphological Characteristics of Idiopathic Inflammatory Myopathies in Juvenile Patients
Source: Cells. 2021 Dec 30;11(1):109. doi: 10.3390/cells11010109 (PMC8750180; doi:10.3390/cells11010109)
Supplement: Supplementary file 1 [file cells-11-00109-s001.zip › Supplemental Table S3 clinical data jIIM subtypes.pdf]

Supplementary Table S3: Clinical data of jIIM patients' subgroups.

	**jIIM**	**DM**	**ASyS Jo-1**	**ASyS PL-7**	**OM**
Number of patients	15 (100 %)	9 (60%)	2 (13%)	2 (13%)	2 (13%)
Age at biopsy in years	8,7 ± 4,4	8,2 ± 4,1	4,5 ± 2,1	12 ± 7,1	12 ± 1,4
Range in years	3 - 17	3 - 16	3 - 6	7 - 17	11 - 13
female	11/15 (73%)	8/9 (89%)	1/2 (50%)	2/2 (100%)	0/2 (0%)
**Skeletal muscle symptoms**	**14/15 (93 %)**	**9/9 (100%)**	**1/2 (50%)**	**2/2 (100%)**	**2/2 (100%)**
Myalgia	11 (73 %)	8 (89%)	0 (0%)	1 (50%)	2 (100%)
Exercise induced myalgia	6 (40 %)	5 (56%)	0 (0%)	1 (50%)	0 (0%)
Proximal weakness	14 (93 %)	9 (100%)	1 (50%)	2 (100%)	2 (100%)
**Skin symptoms**	**15/15 (100 %)**	**9/9 (100%)**	**2/2 (100%)**	**2/2 (100%)**	**2/2 (100%)**
Dry skin	6 (40 %)	3 (33%)	0 (0%)	1 (50%)	2 (100%)
Exanthema	3 (20 %)	2 (22%)	0 (0%)	1 (50%)	0 (0%)
Erythema	4 (27 %)	3 (33%)	0 (0%)	1 (50%)	0 (0%)
Butterfly rash	5 (33 %)	3 (33%)	0 (0%)	1 (50%)	1 (50%)
Redness, livid colouration	8 (53 %)	5 (56%)	2 (100%)	0 (0%)	1 (50%)
Raynaud's phenomenon	1 (7 %)	0 (0%)	0 (0%)	0 (0%)	1 (50%)
Gottron's papules	8 (53 %)	3 (33%)	2 (100%)	2 (100%)	1 (50%)
Nail fold changes	1 (7 %)	1 (11%)	0 (0%)	0 (0%)	0 (0%)
Calcinosis	4 (27 %)	2 (22%)	1 (50%)	0 (0%)	1 (50%)
**Accompanying symptoms**	**15/15 (100 %)**	**9/9 (100%)**	**2/2 (100%)**	**2/2 (100%)**	**2/2 (100%)**
Fever	3 (20 %)	2 (22%)	0 (0%)	1 (50%)	0 (0%)
Difficulty swallowing	1 (7 %)	1 (11%)	0 (0%)	0 (0%)	0 (0%)
Morning stiffness	1 (7 %)	1 (11%)	0 (0%)	0 (0%)	0 (0%)
Arthritis	1 (7 %)	1 (11%)	0 (0%)	0 (0%)	0 (0%)
Weight gain	2 (13 %)	2 (22%)	0 (0%)	0 (0%)	0 (0%)
Weight loss	1 (7 %)	0 (0%)	1 (50%)	0 (0%)	0 (0%)
Night sweat	1 (7 %)	1 (11%)	0 (0%)	0 (0%)	0 (0%)
Oedema	3 (20 %)	3 (33%)	0 (0%)	0 (0%)	0 (0%)
Lymphadenopathy	1 (7 %)	0 (0%)	0 (0%)	0 (0%)	1 (50%)
Fatigue / concentration	7 (47 %)	3 (33%)	1 (50%)	1 (50%)	1 (50%)
difficulties	1 (7 %)	1 (11%)	0 (0%)	0 (0%)	0 (0%)

Loss of appetite/ rejection to drink	5 (33 %)	3 (33%)	1 (50%)	1 (50%)	0 (0%)
Sadness / mood slump/ mood swings	3 (20 %)	3 (33%)	0 (0%)	0/2 (0%)	0/2 (0%)
Social withdrawal					
**Extramuscular symptoms**	**6/15 (40 %)**	**5/9 (56%)**	**1/2 (50%)**	**0/2 (0%)**	**0/2 (0%)**
Pulmonary restriction	2 (13 %)	2 (22%)	0 (0%)	0 (0%)	0 (0%)
Pneumonia	1 (7 %)	1 (11%)	0 (0%)	0 (0%)	0 (0%)
Cardial involvement	4 (27 %)	3 (33%)	1 (50%)	0 (0%)	0 (0%)
**Comorbidities**	**5/15 (33 %)**	**3/9 (33%)**	**0/2 (0%)**	**1/2 (50%)**	**1/2 (50%)**
Cystic fibrosis	1 (7 %)	0 (0%)	0 (0%)	1 (50%)	0 (0%)
Factor-V-Leiden mutation	1 (7 %)	1 (11%)	0 (0%)	0 (0%)	0 (0%)
Hypothyroidism	1 (7 %)	0 (0%)	0 (0%)	0 (0%)	1 (50%)
Steatosis hepatis	1 (7 %)	1 (11%)	0 (0%)	0 (0%)	0 (0%)
HLA-B27 enthesitis	1 (7 %)	1 (11%)	0 (0%)	0 (0%)	0 (0%)
**Laboratory results**	**15/15**	**9/9**	**2/2**	**2/2**	**2/2**
Creatine kinase (CK) ↑	**(100%)**	**(100%)**	**(100%)**	**(100%)**	**(100%)**
Lactate dehydrogenase	14 (93 %)	8 (89%)	2 (100%)	2 (100%)	2 (100%)
(LDH) ↑	11 (73 %)	7 (78%)	1 (50%)	2 (100%)	1 (50%)
GOT ↑	11 (73 %)	6 (67%)	2 (100%)	2 (100%)	1 (50%)
GPT ↑	7 (47 %)	3 (33%)	1 (50%)	2 (100%)	1 (50%)
	**11/15**	**5/9 (56%)**	**2/2**	**2/2**	**2/2**
**MRI skeletal muscle**	**(73 %)**	0 (0%)	**(100%)**	**(100%)**	**(100%)**
Normal	2 (18 %)	4 (44%)	0 (0%)	1 (50%)	1 (50%)
Oedema	6 (54 %)	2 (22%)	1 (50%)	0 (0%)	1 (50%)
Enhancement of contrast	4 (36 %)		1 (50%)	1 (50%)	0 (0%)
medium / signal alterations		4/9 (44%)			
Compatible with myositis	8/11 (73 %)	**6/9 (67%)**	2/2	1/2 (50%)	1/2 (50%)
**Musculosceletal**	**9/15 (60 %)**	2/9 (22%)	(100%)	**1/2 (50%)**	**0/2 (0%)**
**Ultrasound**	3/9 (33 %)	4/9 (44%)	**2/2**	1/2 (50%)	0/2 (0%)
Oedema	6/9 (67 %)		**(100%)**	1/2 (50%)	0/2 (0%)

Enhancement of		3/9 (33%)	0/2 (0%)		
echogenicity / signal	6/9 (67 %)	**7/9 (78%)**	1/2 (50%)	1/2 (50%)	0/2 (0%)
alterations	**11/15**	7/9 (78%)		**2/2**	**2/2**
Compatible with myositis	**(73 %)**		2/2	**(100%)**	**(100%)**
**Electromyography (EMG)**	8/11 (72 %)	0/9 (0%)	(100%)	0/2 (0%)	1/2 (50%)
Myopathic, compatible with			**0/2 (0%)**		
myositis	3/11 (27 %)		0/2 (0%)	2/2	1/2 (50%)
Unspecific sign				(100%)	
			0/2 (0%)		
