# Supplementary material for: Morphological Characteristics of Idiopathic Inflammatory Myopathies in Juvenile Patients
Source: Cells. 2021 Dec 30;11(1):109. doi: 10.3390/cells11010109 (PMC8750180; doi:10.3390/cells11010109)
Supplement: Supplementary file 1 [file cells-11-00109-s001.zip › Supplemental Table S4 Clinical diagnostics.pdf]

Supplementary Table S4: Clinical diagnostic data of patients with Jiim.

	Diagnosis	MRI	EMG		Ultrasound
			Location (muscle)		
**P1**	DM	Compatible with myositis, especially in straight abdominal muscles, autochthonous muscles, gluteal muscles, Mm. quadriceps femoris and lower leg extensors	deltoid	Moderate spontaneous activity, low amplitude, short polyphasic potentials, compatible with DM	Compatible with MRI, oedema on the affected muscles with hyperintensity
**P2**	DM	Mild oedema in the thigh musculature with mild enhancement after application of contrast medium	deltoid, vastus lateralis	Myopathic EMG with small and short polyphasic potentials. No signs of spontaneous activity	Coarsed texture with increased echogenicity
**P5**	DM	n.a.	deltoid, triceps brachii, biceps brachii, vastus lateralis and medialis	Short polyphasic potentials, pathologic spontaneous activity compatible with inflammatory muscle disease	n.a.
**P10**	DM	n.a.	n.a.	n.a.	Compatible with myositis
**P11**	DM	n.a.	n.a.	n.a.	n.a.
**P12**	DM	Distinct signal alterations and oedema compatible with DM	deltoid	Strong myopathic pattern	n.a.
**P15**	DM	Symmetric oedema compatible with DM	deltoid	Pronounced myopathic process with moderate spontaneous activity	Signal alterations in the lower leg muscles.
**P4**	DM, (NXP-2)	Symmetric proximal oedema compatible with myositis	Deltoid, vastus lateralis	Myopathic EMG compatible with myositis. Short polyphasic potentials, low amplitude, and early recruitment	Inhomogeneous proximal oedema of the affected muscles
**P13**	DM, (NXP-2)	n.a.	deltoid	Myopathic EMG compatible with DM/PM	Inhomogeneous signal alterations compatible with myositis
**P8**	ASyS (Jo-1)	Symmetric oedema and signal increases in all examined muscles especially Mm. quadriceps femoris, compatible with myositis	n.a.	n.a.	Patchy signal increases and loss of original muscle structure compatible with myositis
**P14**	ASyS (Jo-1)	Pronounced pathology of the muscle fibres compatible with an immune-mediated myositis like DM	n.a.	n.a.	Inhomogeneous patches compatible with myositis

**P6**	ASyS (PL-7)	Ubiquitous symmetric signal increases, compatible with DM	deltoid	Light myopathic signs not typically like DM/PM	n.a.
**P9**	ASyS (PL-7)	Normal	deltoid	Few myopathic signs, not typical of myositis	Signal increases and oedema in proximal muscles compatible with DM/PM
**P3**	OM (SLE)	Normal	deltoid	Few spontaneous activity, short polyphasic potentials and low amplitude compatible with myopathy.	n.a.
**P7**	OM (SLE)	Patchy oedema in M. vastus lateralis and M. rectus femoris compatible with myositis.	deltoid	Light myopathic signs not typical like DM/PM.	n.a.

Magnetic resonance imaging ; EMG= Electromyography, DM= Dermatomyositis, ASyS= Antisynthetase Syndrome, OM= Overlap-Myositis;

SLE= systemic lupus erythematosus
