# Supplementary material for: Morphological Characteristics of Idiopathic Inflammatory Myopathies in Juvenile Patients
Source: Cells. 2021 Dec 30;11(1):109. doi: 10.3390/cells11010109 (PMC8750180; doi:10.3390/cells11010109)
Supplement: Supplementary file 1 [file cells-11-00109-s001.zip › Supplemental Table S5 Laboratory data.pdf]

Supplementary Table S5: laboratory data.

	Diagnosis	Gender	Age at biopsy (years)	CK initial (U/l)	CK max (U/l)	LDH (U/l)	GOT (U/l)	GPT (U/l)	ANA	dsDNS (U/ml)	MSA	MAA	Other antibodies
**P1**	DM	m	9	3.697	3.697	746	256	138	<1:80	-	-	-	-
**P2**	DM	f	5	177	177	336	norm	norm	1:160	-	-	-	-
**P5**	DM	f	13	239	239	423	28	norm	n.a.	n.a.	n.a.	n.a.	n.a
**P10**	DM	f	3	10.696	20.893	1152	951	336	<1:80	-	n.a.	n.a.	n.a.
**P11**	DM	f	5	903	903	norm	98	norm	n.a	n.a.	n.a.	n.a.	n.a.
**P12**	DM	f	6	775	1245	415	68	norm	1:640	-	-	-	-
**P15**	DM	f	8	16.384	16.384	1.505	633	193	<1:80	-	-	-	-
**P4**	DM	f	9	1454	1.454	525	norm	norm	1:160	-	NXP-2	PM75	-
**P13**	DM	f	16	norm	norm	norm	norm	norm	1:640	-	NXP-2	-	DFS70
**P8**	ASyS	m	6	17.973	17.973	398	84	norm	1:320	-	Jo-1	-	-
**P14**	ASyS	f	3	9.574	9.574	norm	412	1320	>1:10240	-	Jo-1	-	-
**P6**	ASyS	f	7	2.894	5.200	498	143	110	1:100	-	PL-7	Ro52	-
**P9**	ASyS	f	17	6.700	7.142	867	1159	321	<1:80	-	PL-7	-	-
**P3**	OM	m	13	886	886	341	83	50	>1:10240	206,54	-	-	nRNP/Sm-AK 9,2<br>Sm-AK 1,9
**P7**	OM	m	11	237	237	norm	norm	norm	>1:10240	-	-	-	nRNP/Sm-AK 8,9

n.a. not analysed; - negative; laboratory data are compared to age dependent normative values
