# Supplementary material for: Morphological Characteristics of Idiopathic Inflammatory Myopathies in Juvenile Patients
Source: Cells. 2021 Dec 30;11(1):109. doi: 10.3390/cells11010109 (PMC8750180; doi:10.3390/cells11010109)
Supplement: Supplementary file 1 [file cells-11-00109-s001.zip › Supplemental Table S6 Morphology controls.pdf]

Supplementary Table S6: Morphological Scoring of the controls.

	Diagnosis	VAS-Score	HE						COX-SDH	Alkaline phosphatase
			Perifascicular atrophy	Punched-out-vacuoles	Necrosis	Regeneration	Oedema	Inflammation	COX-negative fibers	Upregulation
**C1**	control	0	0	0	0	0	0	0	0	0
**C2**	control	0	0	0	0	0	0	0	0	0
**C3**	control	0	0	0	0	0	0	0	0	0
**C4**	control	0	0	0	0	0	0	0	0	0
**C5**	control	0	0	0	0	0	0	0	0	0
**C6**	control	0	0	0	0	0	0	0	0	0
**C7**	control	0	0	0	0	0	0	0	0	0

	Diagnosis	Inflammation and immunoactivation						Complement deposits (C5b9)		MxA 2	Regeneration		Sarcomere disruption	Angiogenesis		Autophagy	
		MHC1	MHC2	CD3	CD8	CD20	CD68	sarcolemma	capillaries		CD56	MHCd	Myotilin	CD31 rarefication	VEGF	LC3	p62
**C1**	control	0	0	0	0	0	0	0	0	0	0	0	0	0	0	0	0
**C2**	control	0	0	0	0	0	0	0	0	0	0	0	0	0	0	0	0
**C3**	control	0	0	0	0	0	0	0	0	0	0	0	0	0	0	0	0
**C4**	control	0	0	0	0	0	0	0	0	0	0	0	0	0	0	0	0
**C5**	control	0	0	0	0	0	0	0	0	0	0	0	0	0	0	0	0
**C6**	control	0	0	0	0	0	0	0	0	0	0	0	0	0	0	0	0
**C7**	control	0	0	0	0	0	0	0	0	0	0	0	0	0	0	0	0

	Diagnosis	Myofibrillar structure			Mitochondria			Endomysial capillaries	Nuclear inclusions
		Sarcomere structure	Z-line-alteration	Glycogen depositis	Aggregation	Polymorphism	Inclusions	Inclusions in endothelial cells or pericytes/ number of viewed vessels	
**C1**	control	0	0	0	0	0	0	0/10	
**C2**	control	0	0	0	0	0	0	0/10	
**C3**	control	0	0	0	0	0	0	0/10	
**C4**	control	0	0	0	0	0	0	0/10	
**C5**	control	0	0	0	0	0	0	0/10	
**C6**	control	0	0	0	0	0	0	0/10	
**C7**	control	0	0	0	0	0	0	0/10	
